# Placebo and nocebo effects on pain through the lens of the predictive brain: Neurobiological mechanisms and translational implications

**DOI:** 10.1113/EP093418

**Published:** 2026-08-28

**Authors:** Denisa Adina Zamfira, Tommaso Fassina, Kamil Zaworski, Elisa Carlino, Giacomo Rossettini

**Affiliations:** ^1^ Department of Neurosciences “Rita Levi Montalcini” University of Turin Turin Italy; ^2^ Department of Physiotherapy John Paul II University Biała Podlaska Poland; ^3^ Laboratorio di Neurobiologia Istituto Auxologico Italiano ‐ IRCCS Ospedale S. Giuseppe Piancavallo, Oggebbio Verbano‐Cusio‐Ossola Italy; ^4^ School of Physiotherapy University of Verona Verona Italy; ^5^ Department of Physiotherapy Faculty of Medicine, Health and Sports Universidad Europea de Madrid Villaviciosa de Odón Spain; ^6^ Centre for PAIn Research Health Sciences University Bournemouth UK

**Keywords:** expectations, nocebo hyperalgesia, pain, placebo analgesia, predictive processing

## Abstract

Placebo and nocebo effects on pain provide a model for investigating how cognitive and contextual factors modulate the physiological processes underlying nociception and its regulation. However, existing accounts remain fragmented and lack a unifying mechanistic framework linking brain, behaviour and autonomic responses. Here, we synthesize evidence from neuroimaging, electrophysiology, pharmacological and experimental paradigms within a predictive processing framework, conceptualizing pain as an inferential process arising from the integration of top‐down predictions and bottom‐up nociceptive input weighted by their precision. Across studies, placebo analgesia is consistently associated with increased engagement of prefrontal–cingulate circuits and descending modulatory pathways, including periaqueductal grey and rostral ventromedial medulla, alongside activation of opioidergic, dopaminergic and cannabinoid systems. In contrast, nocebo hyperalgesia involves increased activity in limbic–interoceptive networks, recruitment of stress‐related systems and cholecystokinin‐mediated facilitation of nociceptive transmission. Within this framework, placebo and nocebo effects emerge as precision‐dependent modulations of predictive signals that shape both central processing and peripheral physiological outputs, including autonomic and neuroendocrine responses. We further identify methodological approaches to operationalize priors, precision and prediction errors, and discuss how inter‐individual variability may reflect differences in computational and physiological phenotypes. This integrative review offers a mechanistic bridge between cognitive context and physiological regulation, offering a novel perspective on how endogenous predictions can be harnessed to enhance treatment efficacy and mitigate adverse outcomes in clinical practice.

## Abstract (highlights)

- **What is the topic of this review?**
  This review examines placebo analgesia and nocebo hyperalgesia through a predictive processing framework, integrating evidence from neuroimaging, electrophysiology and pharmacology.
- **What advances does it highlight?**
  This review proposes an integrative account in which placebo and nocebo effects can be understood as precision‐weighted predictive processes linking brain circuits, neuromodulatory systems and physiological regulation. It also highlights methodological strategies for operationalizing priors, precision and prediction errors, and discusses how these mechanisms may inform clinical translation.

## 1 INTRODUCTION

Pain represents one of the most informative models for investigating placebo and nocebo effects, as its experience is not solely determined by nociceptive input but is intrinsically shaped by contextual, cognitive and affective factors (Belleï‐Rodriguez & Colloca, 2025). Manipulations of expectation, prior experience, learning, conditioning and treatment context can produce substantial changes in pain perception and related physiological responses, even in the absence of alterations in the physical properties of nociceptive input (Colloca, 2020). For this reason, pain has been extensively adopted as an experimental and clinical paradigm to examine how the brain actively constructs subjective experience and modulates treatment outcomes (Rodrigues et al., 2025).

In experimental and clinical pain research, placebo and nocebo effects are commonly defined as decreases (placebo analgesia) or increases (nocebo hyperalgesia) in pain perception that cannot be attributed to the specific pharmacological or physical properties of a treatment, but rather to contextual and psychosocial factors surrounding its administration (Colloca, 2024; Theodosis‐Nobelos et al., 2021). Such effects emerge from the general neurocognitive architecture of the brain, through which expectations, learning and contextual information interact with sensory processing to shape pain experience (Büchel et al., 2014; Carlino & Vase, 2018; Chen et al., 2024; Rossettini et al., 2020). However, existing explanatory models have largely focused on isolated components, such as expectancy, conditioning or learning, without providing an integrative account of how these processes are coordinated across hierarchical levels of the nervous system (Atlas, 2023; Bąbel, 2020; Bajcar & Bąbel, 2024). As a result, findings from neuroimaging, neurophysiological and neurochemical studies are often interpreted in parallel rather than within a unified mechanistic framework, limiting both theoretical integration and clinical translation.

The predictive processing framework offers such a unifying perspective by conceptualizing pain as an inferential process rather than a direct readout of nociceptive input (Kaptchuk et al., 2020). This account integrates predictive coding and Bayesian inference into brain function, postulating that when estimating the probability of any event, the brain uses the current evidence for that event but also prior knowledge (e.g., past experiences, beliefs, expectations) about the event (Aitchison & Lengyel, 2017; Clark, 2013; Pouget et al., 2013). Within this framework, pain can be conceptualized as emerging from the dynamic interaction between top‐down priors (the brain's pre‐existing internal representations about bodily states) and bottom‐up sensory signals, whose relative influence depends on their assigned precision (Büchel et al., 2014; Grahl et al., 2018; Yoshida et al., 2013). While precision represents the inverse of variance or uncertainty of a signal or belief (i.e., its weight or implicit influence in shaping perception), confidence refers to its metacognitive counterpart (i.e., subjective, conscious feeling) (Camerone et al., 2025; Mulders et al., 2023). Experimentally, precision can be manipulated through changes in stimulus uncertainty, reliability or variability, as well as through implicit or explicit expectation‐conditioning procedures, thereby altering the relative influence of priors and sensory evidence during perceptual inference (Augustat et al., 2024; Brown et al., 2008; Jung et al., 2017). Confidence has received considerably less empirical attention and its computational role within predictive processing remains a matter of ongoing debate, particularly in the context of pain. Recent evidence (Camerone et al., 2025) suggests that both the magnitude of pain expectancy and the confidence with which it is held jointly contribute in determining placebo hypoalgesia and nocebo hyperalgesia. Statistical‐learning studies (Onysk et al., 2024) further indicate that confidence can emerge from internal model formation even in the absence of explicit cues, suggesting that it may reflect the accumulated evidence supporting an individual's internal predictive model in ambiguous environments. At the clinical level, confidence of treatment expectations has been shown to predict therapeutic outcomes (Müller‐Schrader et al., 2023). These findings highlight the need to better elucidate the independent and joint contributions of expectancy and confidence in shaping pain perception, thereby advancing computational models of predictive processing and improving our understanding of inter‐individual variability in treatment responsiveness.

These inferential processes are implemented across distributed neural circuits and neuromodulatory systems, allowing contextual cues to shape nociceptive information processing. Incoming sensory signals are hypothesized to generate prediction errors that update or reinforce existing predictions, thereby continuously shaping pain perception (Büchel et al., 2014). From this perspective, placebo and nocebo effects can be understood as systematic modulations of pain‐related predictions and their precision. Placebo analgesia has been proposed to arise when predictions of relief attenuate the impact of nociceptive prediction errors, engaging descending modulatory pathways that reduce pain experience (Ongaro & Kaptchuk, 2019). In contrast, nocebo hyperalgesia can be interpreted as reflecting the amplification of nociceptive prediction errors driven by highly precise predictions of threat or harm, leading to increased pain and heightened physiological stress responses (Rossettini et al., 2022). Importantly, nocebo effects should not be regarded simply as the negative counterpart of placebo effects, but rather as expressions of partially distinct predictive and neurobiological processes within the broader architecture of pain regulation.

In this review, we adopt the predictive processing framework as a theory‐driven lens to examine the neurobiological mechanisms underlying placebo and nocebo effects on pain. By integrating evidence from neuroimaging and electrophysiology approaches, we aim to elucidate how predictive processes are implemented across pain‐related neural circuits and neuromodulatory systems. We specifically distinguish the predictive mechanisms supporting placebo analgesia from those driving nocebo hyperalgesia and discuss how these mechanisms can be systematically engaged or mitigated in clinical contexts. Finally, we address key methodological challenges for investigating predictions, precision and prediction error signalling in pain research.

## 2 PAIN AS A PREDICTIVE PERCEPT: CORE PRINCIPLES OF PREDICTIVE PROCESSING

Within the predictive processing framework, pain is no longer conceptualized as a passive perceptual sub‐modality of the sense of touch (Craig, 2003). Instead, it is an embodied and homeostatic phenomenon emerging from brain's hierarchical generative models that subserve individuals’ attempts to plan and implement self‐regulation strategies and adaptive reactions toward the environment (Atlas, 2023; Craig, 2003; Eccleston, 2018; Mancini et al., 2022; Ongaro & Kaptchuk, 2019; Wiech, 2016). When processing noxious stimuli, predictive processing theories propose that the brain actively anticipates their occurrence and interprets them through a dynamic interplay between top‐down priors and bottom‐up sensory evidence (Büchel et al., 2014; Friston, 2010), weighted by their relative precision (Milde et al., 2024), such that the greater the precision of each source, the greater its influence on the final percept (Büchel et al., 2014; Grahl et al., 2018; Yoshida et al., 2013). When the prior precision is high, its influence on the interpretation of the incoming sensory input is maximal (the percept shifts towards the prior), offering an explanation for perceptual biases, including placebo hypoalgesia and nocebo hyperalgesia.

According to predictive processing accounts of brain function, every external or interoceptive noxious input generates an inferential prediction about the incoming experience. This prediction is hypothesized to be based on the integration between the salient contextual and stimulus‐related information, the previously stored experience‐related knowledge and memories, and the information about the actual state of the body (Büchel et al., 2014; Friston, 2010). These (implicit) predictions are tested against the occurrence of an internal or external painful event to verify their resemblance. If the incoming input is coherent with the predicted one, the prediction is proposed to be confirmed. If there is a mismatch, a prediction error signal is generated and propagated to several hierarchical levels using feedforward and feedback projections in order to optimize the parameters of the model (i.e., to update the prior prediction) or to modify the processing or interpretation of the sensory input to better fit the prediction (Eccleston, 2018; Panerai, 2011).

**FIGURE 1:**
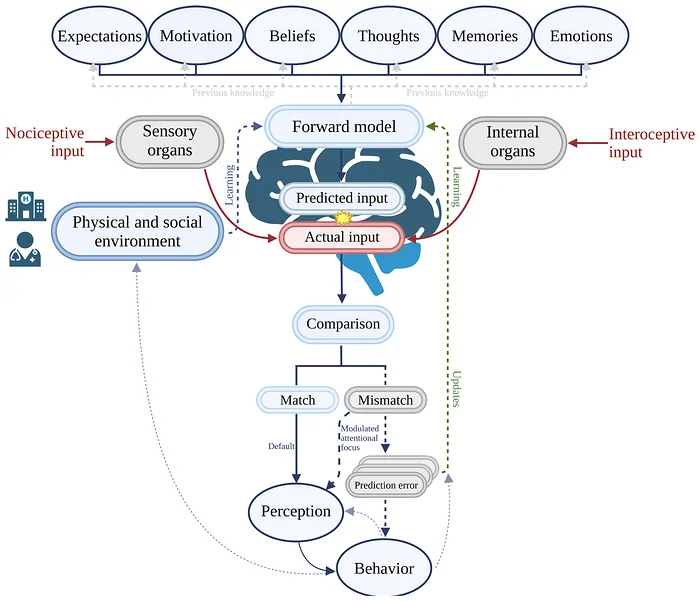
Pain as a Bayesian inference process. Sensory (nociceptive) and interoceptive inputs are integrated with prior beliefs, expectations and memories of past experiences to generate predictions about bodily states (forward model). These predictions are continuously compared with incoming sensory evidence, producing either a match, leading to stable perception, or a mismatch, generating a prediction error signal. Prediction errors drive updating processes through learning, modulating attentional focus and shaping subsequent perception. Physical and social environmental factors further influence this dynamic interplay, highlighting pain as an emergent, context‐dependent inference rather than a passive reflection of sensory signals. Each new experience contributes to the updating of prior beliefs, refining future inferences and dynamically shaping the perception of pain. Blue arrows represent top‐down predictions about the world that are propagated to lower levels of the hierarchy, whereas red arrows represent bottom‐up sensory inputs against which these predictions are compared. Created using BioRender.com.

Predictive processing models posit that priors are updated with each new experience by integrating new information (likelihood), producing a posterior that in turn becomes the new prior. This ongoing process of belief updating (Büchel et al., 2014) is fundamental for shaping future inferences (Wiech, 2016). Moreover, it has been suggested that the brain largely resolves environmental uncertainty through inference and prediction outside conscious awareness. When mismatches cannot be resolved at lower levels of hierarchical processing, ascending afferent signals gain salience and give rise to intrusive conscious sensations that require attentional monitoring and compensatory behaviour (Clark, 2013; Eccleston, 2018; Panerai, 2011) (Figure 1).

At least three reciprocally interconnected large‐scale brain networks have been shown to be involved in these predictive processing operations: the salience network (SN), the default‐mode network (DMN) and the central executive network (CEN) (Menon, 2011). The ventromedial prefrontal cortex, the posterior cingulate cortex, the precuneus and the lateral parietal cortex are part of the DMN, primarily involved in self‐referential mental processes, as well as in monitoring and processing affective and social‐related information (Raichle et al., 2001). This network is crucial for creating mental simulations and expectations (i.e., conscious cognitive states) about future events by integrating personal past experiences (Buckner et al., 2008). The rostral prefrontal cortex, anterior insula, anterior cingulate cortex, amygdala, ventral striatum and substantia nigra are part of the SN and are mainly responsible for detecting, integrating and filtering relevant interoceptive, sensory, environmental and emotional information. The SN has been suggested to contribute to monitoring prediction error signals, and when required, it performs cognitive control, switches the attentional focus toward salient stimuli and engages the CEN to select and drive the more appropriate behavioural responses (Menon, 2015). The CEN comprises the prefrontal cortex and posterior parietal cortex. It plays a prominent role in the active maintenance of salient information in working memory, as well as in planning, problem‐solving, emotion regulation, executive functioning, decision‐making and higher‐order cognitive control. When salient events require high cognitive demand and the selection of an appropriate action to be solved, the CEN network is recruited by the SN, while simultaneously disengaging the DMN, facilitating the information's access to sustained attention, memory and higher‐order cognitive resources (Menon & Uddin, 2010). Moreover, this fronto‐parietal network is believed to update predictions of sensory stimuli while generating prediction errors (Schwartenbeck et al., 2016).

**FIGURE 2:**
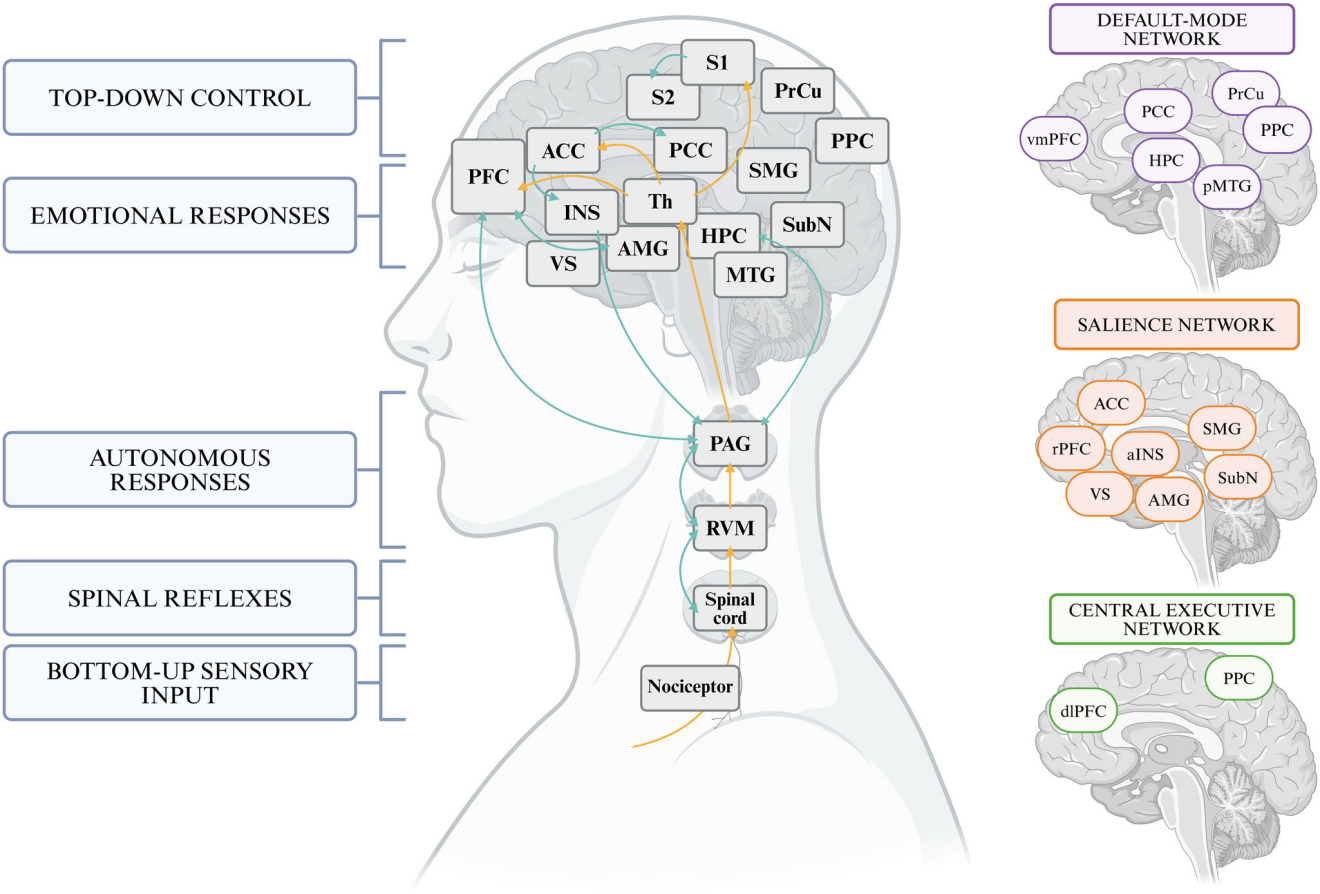
Neural underpinnings of pain perception and modulation. Schematic representation of the distributed neural network supporting pain processing and its top‐down modulation. Nociceptive signals originating from peripheral receptors ascend via the spinal cord to the thalamus (Th), which relays sensory information to primary (S1) and secondary (S2) somatosensory cortices for the encoding of sensory‐discriminative features of pain. Parallel projections engage limbic and salience‐related regions, including the insula (INS), anterior and posterior cingulate cortex (ACC and PCC), amygdala (AMG), and hippocampus (HPC), supporting the affective, motivational dimensions of pain. The prefrontal cortex (PFC) exerts top‐down control over these regions, integrating cognitive and contextual information to shape pain perception. Descending modulatory pathways, involving the periaqueductal gray (PAG) and rostral ventromedial medulla (RVM), regulate nociceptive transmission at the spinal level, enabling dynamic amplification or inhibition of pain signals. Created using BioRender.com.

The hierarchical organization of predictive processing implies that cognitive and affective states (such as expectations of relief or harm) deeply influence lower‐level nociceptive processing. Thus, the magnitude of the subjective pain experience is not linearly related to the amount of noxious stimulation (e.g., the number of nociceptors activated) and to the duration of the stimulation itself, but is shaped by the precision of internal predictions and contextual cues. The experience of pain can, in fact, occur in the absence of noxious stimuli (e.g., during hypnosis suggestion or nocebo procedures) and the nociception process could activate neural responses without necessarily leading to a conscious pain experience (Atlas, 2023; Carlino et al., 2014; Lee et al., 2009; Legrain et al., 2011; Loeser & Treede, 2008; Nickel et al., 2017). Moreover, expectations, which are formed by prior experiences, verbal suggestions and the therapeutic context, are hypothesized to strongly modulate prediction errors, leading to significant alterations in pain perception (Atlas, 2023). For instance, when expectations of pain relief are induced, the brain may downweight nociceptive prediction errors, attenuating the experience of pain (placebo analgesia). In contrast, negative expectations and threatening cues can be conceptualized as increasing the relative weighting of nociceptive predictions, increasing pain perception (nocebo hyperalgesia) (Ongaro & Kaptchuk, 2019; Pagnini et al., 2023). Moreover, clinical evidence shows that the predictive brain is sensitive not only to verbal influences, but also to subtle environmental and interpersonal cues, which can shape priors about pain controllability and threat (Rossettini et al., 2023; Rossettini et al., 2018). Thus, placebo and nocebo responses can be conceived as an endogenous modulation of the nociceptive activity, based on the precision of internal predictions resulting in an attenuation or amplification of the sensory input (Büchel et al., 2014; Grahl et al., 2018; Milde et al., 2024; Yoshida et al., 2013) (Figure 2).

## 3 NEUROBIOLOGY OF PLACEBO ANALGESIA IN THE PREDICTIVE BRAIN

Placebo analgesia can be interpreted as a shift in the brain's generative model in which an ongoing hypothesis of pain reduction becomes the most precise explanation for incoming sensory evidence. Such a hypothesis may increase the precision of top‑down predictions of relief, potentially allowing them to modulate the perception of nociceptive and interoceptive signals (Grahl et al., 2018). Evidence from open–hidden administration paradigms shows that symptom‐relieving drugs produce markedly smaller effects when administered covertly than when patients are aware of receiving them (Benedetti et al., 2011). Within a predictive processing framework, this finding is consistent with the idea that awareness of treatment administration and the associated therapeutic context – including explicit expectations of benefit, rituals and other contextual cues – may strengthen prior beliefs about improvement, thereby influencing the top‐down processing of bodily signals. Under such strengthened priors, even minimal interoceptive fluctuations are perceptually interpreted as signs of healing, and the resulting experience of relief may become self‑fulfilling as prediction errors are minimized in favour of the relief hypothesis (Ongaro & Kaptchuk, 2019). Contextual cues further shape these priors: for instance, inert treatments described as costly elicit stronger beneficial effects than those presented as inexpensive (Kam‐Hansen et al., 2014), and observing others benefiting from a treatment enhances one's own placebo response through social learning (Colloca & Benedetti, 2009).

**FIGURE 3:**
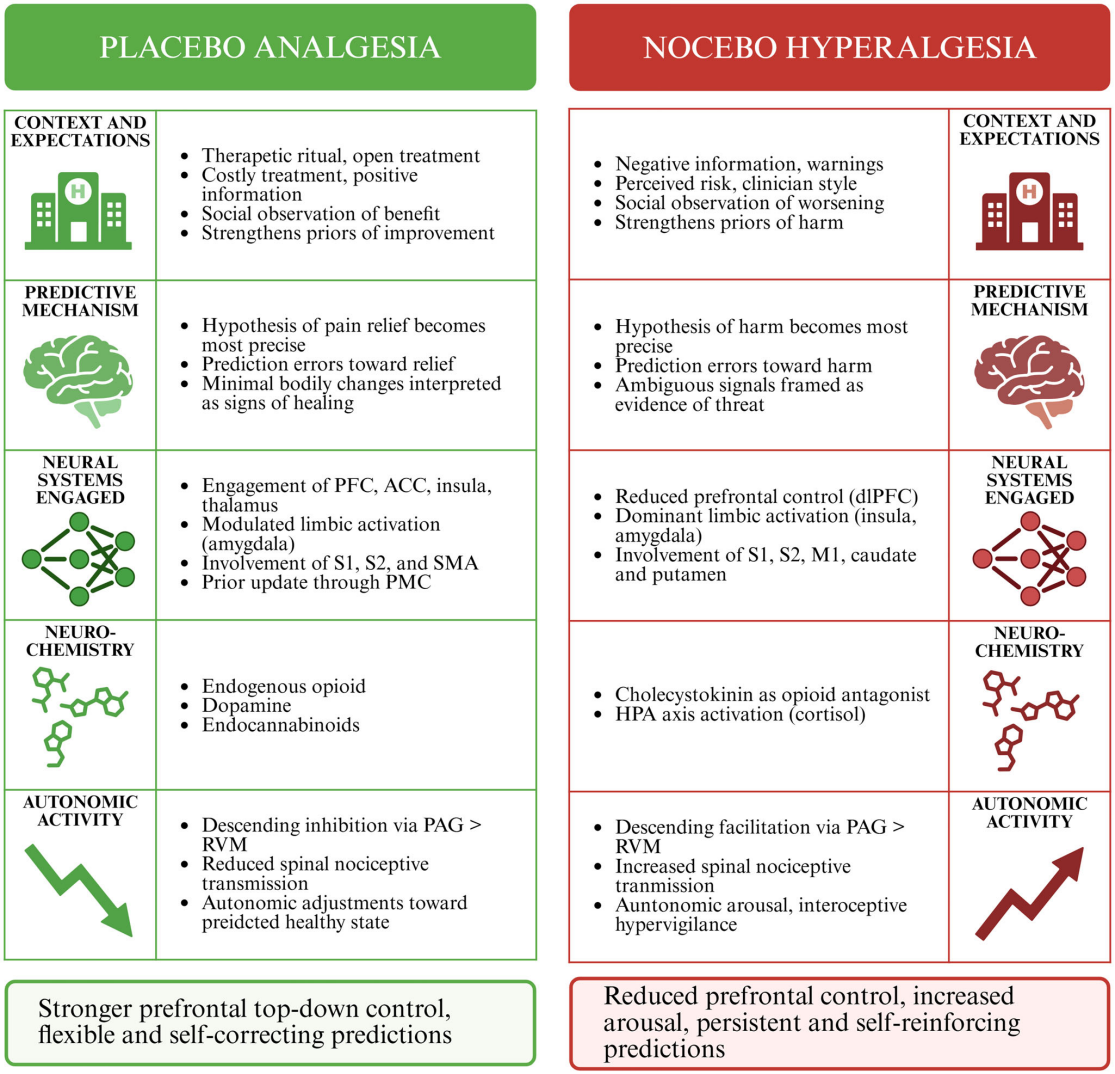
Cognitive and neurobiological determinants of placebo analgesia and nocebo hyperalgesia. Schematic representation of the cognitive and neurobiological mechanisms underlying placebo analgesia and nocebo hyperalgesia within the predictive processing framework. Contextual information, prior and expectations can shape predictions of relief or harm, influencing the relative precision assigned to incoming sensory signals. During placebo analgesia, prior expectations of relief become dominant, promoting top‐down modulation by prefrontal and cingulate regions and engagement of somatosensory, insular, thalamic and premotor networks. These processes are supported by endogenous opioids, dopamine and endocannabinoids and contribute to descending inhibition through the periaqueductal grey (PAG) and rostral ventromedial medulla (RVM), reducing nociceptive transmission. In contrast, nocebo hyperalgesia emerges when negative priors (i.e., expectations of harm or symptom worsening) become dominant, biasing perception toward threat and amplifying pain‐related processing. This state is associated with reduced prefrontal control, increased engagement of limbic and salience‐related regions, activation of the hypothalamic–pituitary–adrenal (HPA) axis and cholecystokinin‐mediated mechanisms, and descending pain facilitation via the PAG–RVM pathway, resulting in enhanced nociceptive transmission and autonomic arousal. Areas in red squares indicate relative increases in regional activity or functional engagement, while areas in green squares indicate a relative decrease in brain activity. Created using BioRender.com.

Functional neuroimaging studies have revealed that placebo responses are implemented through a hierarchical recurrent system including cortical (prefrontal cortex, anterior cingulate cortex and anterior insula), subcortical (amygdala, hypothalamus and thalamus), midbrain (periaqueductal grey), medulla (rostral ventromedial medulla) and spinal regions (Amanzio et al., 2013; Büchel et al., 2014; Figure 3). For instance, predictions of low pain intensity involve the contralateral sensorimotor cortex, supplementary motor cortex, dorsal anterior cingulate cortex, bilateral thalamus and posterior insular–opercular cortex, while the signal representing update of priors has been localized in adjacent premotor and parietal cortices (Mancini et al., 2022). The integration of positive expectations (i.e., pain decrease) with nociceptive sensory inputs has been shown to rely on the functional coupling within brain structures processing positive emotion and inhibiting threat response (e.g. medial frontal cortex and hippocampus; Tsai et al., 2024). Furthermore, positron emission tomography studies (Peciña et al., 2014; Scott et al., 2008; Wager et al., 2004; Zubieta et al., 2005) showed the involvement of several neurotransmitter systems (opioidergic, dopaminergic, cannabinoid) responsible for feedback signalling in the hierarchy and for the specification of the priors (or predictions) (Friston, 2010; Skyt et al., 2020; Theodosis‐Nobelos et al., 2021). Electroencephalographic (EEG) evidence confirms that placebo analgesia responses are associated with altered error‐monitoring processes in the dorsolateral prefrontal cortex (Koban et al., 2012). Crucially, transient and reversible inhibition of this area by means of repetitive transcranial magnetic stimulation (rTMS) was found to disrupt placebo responses (Krummenacher et al., 2010), confirming a causal role of this cognitive control region for the implementation of placebo analgesia.

## 4 NEUROBIOLOGY OF NOCEBO EFFECTS: PREDICTIVE AMPLIFICATION OF PAIN

Nocebo hyperalgesia represents the opposite computational shift in the brain's generative model, in which the hypothesis of harm or symptom worsening biases the interpretation of ambiguous sensory input toward increased pain perception (Geuter et al., 2017). In this case, negative contextual factors, including characteristics of the clinician, clinician–patient interaction, information about possible negative outcomes and the broader therapeutic environment, can reinforce the salience of negative expectations (Blasini et al., 2017) and their specific weight in the determination of the final percept. However, it is important to emphasize that nocebo responses are not merely the ‘other face’ of placebo analgesia, as they are generally more robust, persistent and driven by different cognitive and neurobiological underpinnings.

When negative priors are assigned excessive precision, prediction errors that contradict expectations of pain worsening are attenuated or reinterpreted, while those related to an increased pain experience are maximized. This has been proposed to lead to a self‐reinforcing loop in which ambiguous nociceptive and interoceptive signals are preferentially framed as evidence of bodily threat (Geuter et al., 2017; Ongaro & Kaptchuk, 2019). For instance, the simple verbal suggestion of increased pain can induce hyperalgesia (Benedetti et al., 2007), while conditioning procedures further stabilize threat‐related priors (Tu et al., 2019). Moreover, experimentally manipulating prediction errors (e.g., by increasing stimulus uncertainty or introducing a mismatch between *predicted* and *actual* sensory input) significantly intensifies pain perception (Ishikawa et al., 2025).

Susceptibility to nocebo interventions has been shown to be strongly influenced by individual characteristics (e.g., genetic variants, personality traits), conscious and non‐conscious learning processes, and social observation of pain worsening (Bajcar & Bąbel, 2024; Blasini et al., 2017; Piedimonte et al., 2020; Webster et al., 2016). For instance, individual differences such as anxiety, fear of pain and catastrophizing are positively associated with nocebo responsiveness, suggesting that anticipatory affective states might increase the precision of negative expectations (Corsi & Colloca, 2017; Frisaldi et al., 2015; Tsai et al., 2024), making them more influential in shaping pain perception. Pharmacological reduction of anxiety (e.g., with benzodiazepines) attenuates nocebo hyperalgesia, while the opposite activation of stress‐related neurobiological systems enhances it (Benedetti et al., 1997, 2006). This evidence indicates that during nocebo responses, the organism may enter a state of interoceptive hypervigilance and increased anticipatory anxiety, in which sensory signals are assessed with increased salience and interpreted through a threat‐sensitive prior.

Neuroimaging data show that nocebo effects operate through hierarchical and recurrent systems that partially overlap with those underpinning placebo analgesia but are functionally distinct from it (Figure 3). For instance, differently from predictions of low‐intensity pain stimulation, predictions of high incoming pain are encoded in the bilateral, primary somatosensory and motor regions, secondary somatosensory cortex, caudate, and putamen (Mancini et al., 2022). Functional coupling within anxiety‐processing brain structures (e.g., amygdala and anterior cingulate) has been shown to underpin the integration of negative expectations (i.e., pain worsening) with incoming nociceptive inputs (Tsai et al., 2024). Together with the amygdala, the anterior insula has been suggested to play a central role in assessing threat‐related salience, influencing the resolution of interoceptive prediction errors (Enck et al., 2008; Wiech et al., 2010), while midbrain and brainstem structures, such as the periaqueductal grey and rostral ventromedial medulla, are thought to mediate descending facilitation of nociceptive transmission (Amanzio & Palermo, 2019; Büchel et al., 2014). The parietal cortex, responsible for the computation of the uncertainty of the probabilistic inference of pain, together with the prefrontal cortex, has been suggested to contribute to updating the temporal statistical representation of pain intensity (i.e., continuously tracking the frequency of pain states and their volatility) and to actively use this information for future inferences (Mancini et al., 2022). Interestingly, increased anticipatory anxiety has been shown to impair the updating of expectations via altered prediction error signalling in the anterior cingulate, thus perpetuating negative expectancy effects (Tsai et al., 2024).

Compared to placebo analgesia, nocebo hyperalgesia is associated with reduced engagement of prefrontal regions and a relative dominance of limbic circuits, reflecting a reduced top‐down control over threat‐related predictions and enhanced bottom‐up amplification of nociceptive signals (Colloca, 2017; Geuter et al., 2017). Moreover, this phenomenon has been shown to be neurochemically sustained by the activation of the hypothalamic–pituitary–adrenal axis and the release of cholecystokinin, which acts as a facilitator of descending pain pathways and a functional antagonist to the endogenous opioid system (Benedetti, 1996; Benedetti et al., 2011; Scott et al., 2008).

Several brain regions involved in placebo and nocebo responses, including the anterior insula, anterior cingulate cortex, dorsolateral prefrontal cortex and parietal cortex, are key nodes of the SN, CEN and DMN networks. This overlap suggests that the cognitive operations supported by these large‐scale networks, for example, expectation formation, salience attribution and prediction‐error updating, may constitute the neurocognitive mechanisms through which predictive processing shapes the subjective experience of pain.

## 5 CLINICAL AND TRANSLATIONAL IMPLICATIONS

From a predictive processing perspective, clinical context is crucial in shaping pain‐related priors. Clinical communication does not merely transmit information: it can actively modulate patients’ predictions about symptoms and treatment efficacy (Zaworski et al., 2025, 2026). Verbal suggestions, tone of voice, non‐verbal behaviour and the therapeutic alliance are all factors that contribute to the formation of expectations that influence the evaluation of a nociceptive input (Carlino et al., 2014; Ongaro & Kaptchuk, 2019). Clinician–patient interactions may provide contextual cues that update generative models about anticipated pain and relief. A positive, empathic communication may strengthen analgesic priors, down‐weighting nociceptive prediction errors and facilitating placebo analgesia. On the other hand, an ambiguous or negatively framed communication may increase uncertainty or threat‐related priors, strengthening the nocebo effect (Pagnini et al., 2023). These mechanisms, therefore, play a key role in optimizing both pharmacological and non‐pharmacological interventions. Evidence of the predictive modulation of analgesia comes from open versus covert treatment paradigms, which show that the same analgesic substance produces stronger effects when patients are aware compared to when they are unaware (Benedetti et al., 2003). Within a predictive framework, open administration activates explicit treatment‐related priors that synergize with pharmacodynamic mechanisms, whereas covert administration may remove the contextual cues necessary to activate top‐down analgesic predictions. This interaction suggests that clinical efficacy is partially determined by the expectation‐driven modulations of pain control systems, highlighting that clinical context is a critical determinant of therapeutic outcome.

Materials involving information relevant for the patient, like drug leaflets and consent forms, represent a translational potential source of prediction shaping. Written information about potential side effects can represent a powerful nocebo trigger, especially when framed in a negative or ambiguous way. Studies have shown that informing patients about possible side effects increases their incidence, even when inert substances are administered (Amanzio et al., 2009; Webster et al., 2016). Such information may increase the probability of the prior assigned to negative sensations, increasing body vigilance and the precision of interoceptive prediction errors of harm. Contextual and communicative factors, including written and verbal suggestions, can modulate the outcome of treatments through mechanisms operating at both conscious and unconscious levels, respectively through positive expectations and classical conditioning (Carlino & Benedetti, 2016; Carlino et al., 2014). It follows that framing side effects correctly can recalibrate threat‐related priors, whether emphasizing likelihood or reversibility.

Predictive processing also helps explain how open‐label placebos work. While traditional theories assumed that deception was necessary for placebo analgesia, open‐label studies instead demonstrate that symptom improvement can occur even when patients are explicitly informed about the administration of an inert treatment (Kaptchuk et al., 2010). Open‐label placebos can be conceptualized as a reconfiguration of priors rather than their concealment. The possibility of improvement in the absence of pharmacological action is typically legitimized by emphasizing mind–body interactions and conditioned responses. This reframing may reduce uncertainty, enhance perceived controllability and activate analgesic predictions related to prior learning. Clinically, this suggests that therapeutic benefit can be ethically obtained through a systematic integration of expectations, rather than relying on deception.

## 6 METHODOLOGICAL CHALLENGES AND FUTURE DIRECTIONS

Advancing a mechanistic understanding of placebo and nocebo effects within a predictive processing framework requires approaches that can operationalize priors, precision and prediction errors. As these are latent constructs, future studies should adopt computational models, such as hierarchical Bayesian and active inference frameworks (Büchel et al., 2014), to quantify belief updating and precision weighting from behavioural and physiological data. A challenge is the experimental manipulation of precision. While expectations can be induced through suggestion or conditioning, selectively modulating the relative weight of priors versus sensory input is more difficult (Camerone et al., 2025). Paradigms that vary uncertainty, stimulus reliability, or probabilistic contingencies may help dissociate these components and characterize placebo and nocebo effects as precision‐dependent processes.

Multimodal designs are needed to link levels of analysis. Combining neuroimaging with electrophysiological and autonomic measures can capture both the spatial and temporal dynamics of predictive processing (Kaptchuk et al., 2020). Integrating EEG and functional near infrared spectroscopy allows simultaneous capture of high‐temporal neural dynamics (e.g., event‐related potentials reflecting rapid prediction errors) and localized cortical haemodynamics associated with state transitions (Qiu et al., 2022). Through the use of rTMS, it might be possible to introduce a causal perturbation, enabling targeted activations or inhibitions of specific cortical areas to observe their role within the predictive framework. Peripheral signals such as heart rate variability, skin conductance and pupillary responses serve as explicit somatic markers of interoceptive regulation and predictive precision control (Pezzulo et al., 2019), and may provide information about the physiological cost of prediction errors, helping to map how central priors dynamically modulate bodily states to maintain stability. For instance, heart rate variability may index autonomic flexibility and vagal regulation (Owens et al., 2018; Thayer et al., 2012). Skin conductance responses may reflect sympathetic arousal and salience attribution, and pupillary responses may capture changes in cognitive effort, uncertainty and arousal (Alamia et al., 2019; Xia et al., 2017). Integrating these central and peripheral measures may therefore provide a more comprehensive understanding of how predictive beliefs are generated, updated and translated into subjective experience.

Importantly, inter‐individual variability remains poorly understood (Kaptchuk et al., 2020). Future research should aim to identify computational and physiological phenotypes associated with placebo and nocebo responsiveness, integrating psychological traits. For instance, high autistic traits in the general population have been linked to reduced reliance on priors and increased weighting of sensory information, whereas high schizotypal traits have been linked to alterations in the precision assigned to prior expectations and prediction errors at the expense of that assigned to sensory evidence (Marsicano et al., 2026; Starita & Di Pellegrino, 2018; Tarasi et al., 2023; Teufel et al., 2015). Individual differences along the autism–schizophrenia continuum may represent an index of placebo and nocebo responsiveness. In addition, systematic investigation across biological sex, age groups, and diverse socio‐economic and cultural contexts is essential to capture variability in predictive mechanisms and to enhance the generalizability and translational relevance of findings. This may support stratification and more targeted interventions.

From a translational perspective, predictive mechanisms should be leveraged without deception (Ongaro & Kaptchuk, 2019). Open‐label placebo paradigms suggest that expectation‐based effects can be ethically harnessed, but their mechanisms require further clarification. At the same time, optimizing clinical communication to reduce threat‐related priors may help mitigate nocebo effects. Finally, cross‐species approaches may enhance mechanistic insight. Aligning paradigms across animal and human studies could facilitate the integration of circuit‐level evidence with subjective and contextual aspects of pain. Addressing these challenges will be essential to move toward a quantitatively grounded and mechanistically precise account of placebo and nocebo effects.

## 7 CONCLUSIONS

Placebo and nocebo effects on pain can be interpreted, within a predictive processing framework, as reflecting structured neurobiological processes through which expectations, learning and context shape nociceptive experience. The predictive processing framework provides a potentially unifying account, conceptualizing these effects as precision‐dependent modulations of top‐down predictions and bottom‐up signals implemented across distributed neural and neuromodulatory systems.

Within this perspective, placebo analgesia and nocebo hyperalgesia emerge as partially distinct processes rather than simple opposites, with important implications for both experimental models and clinical practice. Expectations and context are integral components of treatment efficacy, interacting with pharmacological and physical interventions. Integrating computational, neurophysiological and behavioural approaches will be essential to achieve a more mechanistic and physiologically grounded understanding of these effects, and to translate this knowledge into more effective and targeted interventions.

## AUTHOR CONTRIBUTIONS

Denisa Adina Zamfira, Tommaso Fassina, Kamil Zaworski, Elisa Carlino and Giacomo Rossettini contributed to the conception and design of the review, interpretation of the literature, drafting and critical revision of the manuscript. All authors have read and approved the final version of this manuscript and agree to be accountable for all aspects of the work in ensuring that questions related to the accuracy or integrity of any part of the work are appropriately investigated and resolved. All persons designated as authors qualify for authorship, and all those who qualify for authorship are listed.

## CONFLICT OF INTEREST

None declared.

## FUNDING INFORMATION

None.

## GENERATIVE AI STATEMENT

No generative artificial intelligence tools were used in the preparation of this manuscript.

## Data Availability

No new data were generated or analysed in support of this review.
